# Unforeseen Encounters: Managing Intravesical Drains and Secondary Bladder Stones in Clinical Practice

**DOI:** 10.7759/cureus.67220

**Published:** 2024-08-19

**Authors:** Rinki K Meena, Sushanto Neogi, Kishor M, Deepak Ghuliani

**Affiliations:** 1 Department of General Surgery, Maulana Azad Medical College, New Delhi, IND; 2 Department of Surgery, Maulana Azad Medical College, New Delhi, IND

**Keywords:** urethral stones, uremia, urethral stricture, diabetes mellitus, secondary bladder calculus, retained drain

## Abstract

Urinary tract stones predominantly affect the kidneys and ureters, with bladder stones representing a smaller subset. Secondary bladder stones often arise from underlying pathologies such as bladder outlet obstruction, neurogenic bladder dysfunction, or the presence of foreign bodies within the bladder. We present a case of a 54-year-old male with a history of bladder stones and type 2 diabetes mellitus who presented with chronic urinary symptoms and penile swelling. Imaging revealed multiple bladder stones and a periurethral abscess secondary to a retained intravesical drain from a previous cystolithotomy. Surgical intervention included cystolithotomy, removal of the drain and stones, and management of associated urethral strictures. Postoperatively, the patient showed improvement in renal function and resolution of symptoms. This case underscores the importance of vigilant management during drain procedures to prevent complications like retained foreign bodies leading to stone formation and obstructive uropathy.

## Introduction

Urinary tract stones are most commonly found in kidneys and ureters and less common in the urinary bladder. The incidence of urolithiasis is common in developing countries in comparison to developed ones due to improved healthcare facilities and easy access. Bladder stones are rarely due to primary causes, and often diagnosed cases have some underlying pathologies like urinary tract infection, benign prostatic hyperplasia, urethral stricture, or a foreign body that can cause stone formation (secondary stone) [1]. Presentation can be wide ranging from asymptomatic to lower abdominal pain, dysuria, hematuria, or urinary retention [2]. In cases involving intravesical foreign bodies, chronic infections with abscess formation can occur, and in rare instances, this may lead to renal failure. Occurrence of secondary renal stones due to foreign bodies has been documented but the formation of vesical calculi due to unrecognized chronic intravesical drain is seldom seen.

## Case presentation

A 54-year-old male presented to the surgery outpatient department (OPD) with complaints of dribbling of urine and penile swelling for the past six months. The patient developed lower urinary tract symptoms (LUTS) of increased frequency, hesitancy, and sense of bladder fullness for which he didn’t seek medical attention. The patient had a past history of bladder stones for which he underwent cystolithotomy with peri-vesical drain placement 10 years back. The patient is a known case of type 2 diabetes mellitus. On examination, the penile shaft was swollen and soft in consistency, with a hard stone (urethral stone) palpable at the root of the penis. Routine investigations were done for the patient (Table 1).

**Table 1:** Investigations with values and respective normal range

Parameters	Value	Normal range
Hemoglobin	8.2 g/dl	12-15.5 g/dl
Total leucocyte count	3160/cumm	5000-10,000/cumm
Blood urea	113 mg/dl	19-43 mg/dl
Creatinine	4.6 mg/dl	0.66-1.25 mg/dl
Hba1c	5.6%	< 5.5%
Urine routine	40-50 pus cells/hpf, albumin +	nil
Urine culture	No growth	no growth

On the basis of the creatinine value, the estimated glomerular filtration rate (eGFR) was calculated for the patient which was 16ml/min/1.72 m^2^.

**Figure 1:**
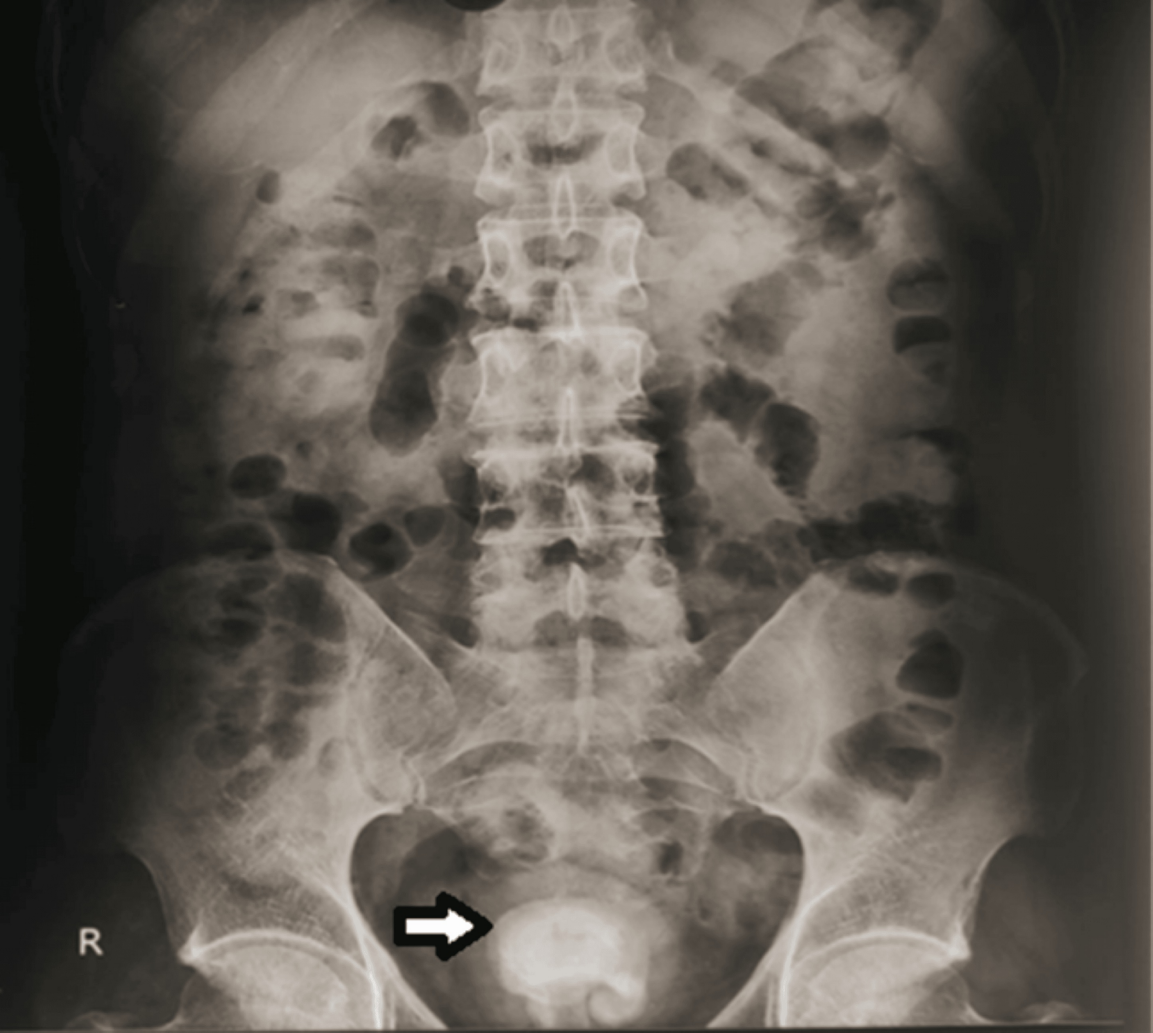
X-ray KUB showing radio-opacity in the bladder region (arrow) KUB: Kidney ureter bladder

X-ray- kidney ureter bladder (KUB) region showed multiple calculi in the bladder region (Figure 1).

Ultrasonography whole abdomen revealed a small right kidney with moderate hydronephrosis with cortical thinning secondary to obstructive uropathy in the left kidney and few calculi in the urinary bladder largest of size 3.4 cm.

**Figure 2:**
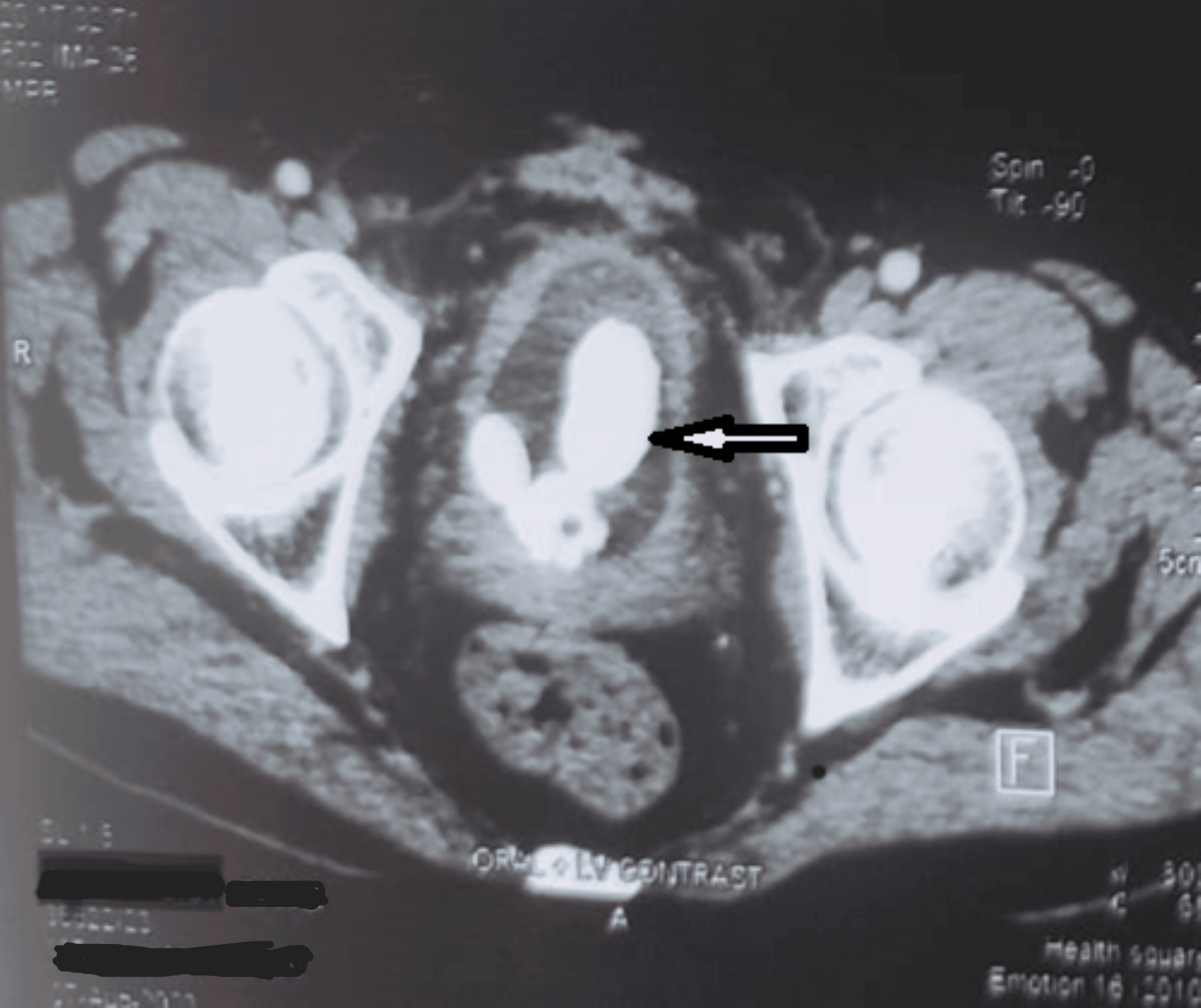
CT film showing intravesical multiple calculi

The contrast-enhanced computed tomography (CECT) abdomen cross-sectional view at the level of the pelvis showing bladder displayed vesical multiple large calculi with drain (Figure 2). 

**Figure 3:**
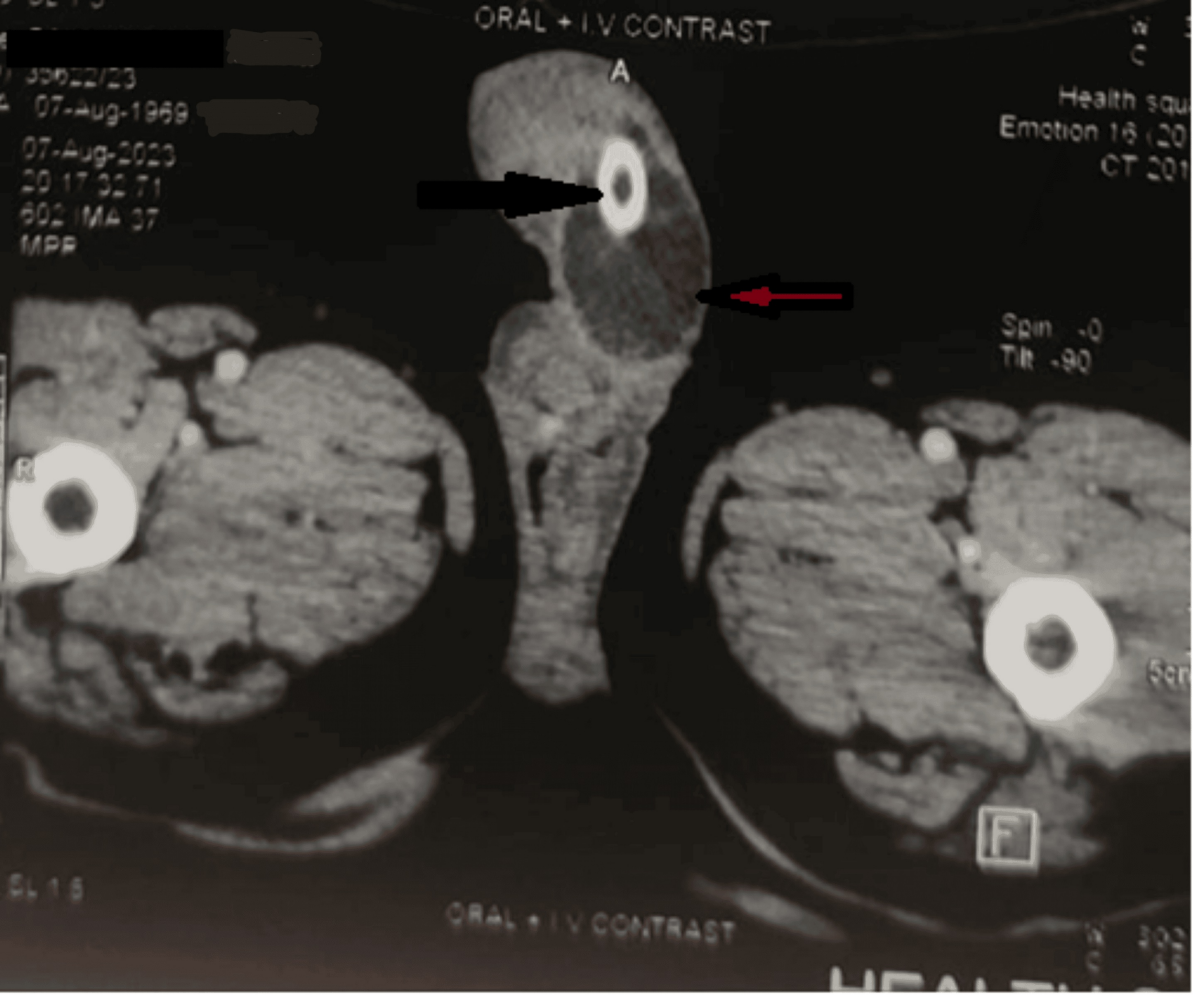
CT film showing drain in posterior urethral (black arrow) with periurethral collection (red arrow)

The CECT abdomen cross-sectional view at the level of the pelvis showing the urethra displayed multiple calculi extending along the posterior urethra, with the largest measuring 2 cm and accompanied by a periurethral collection (Figure 3).

**Figure 4:**
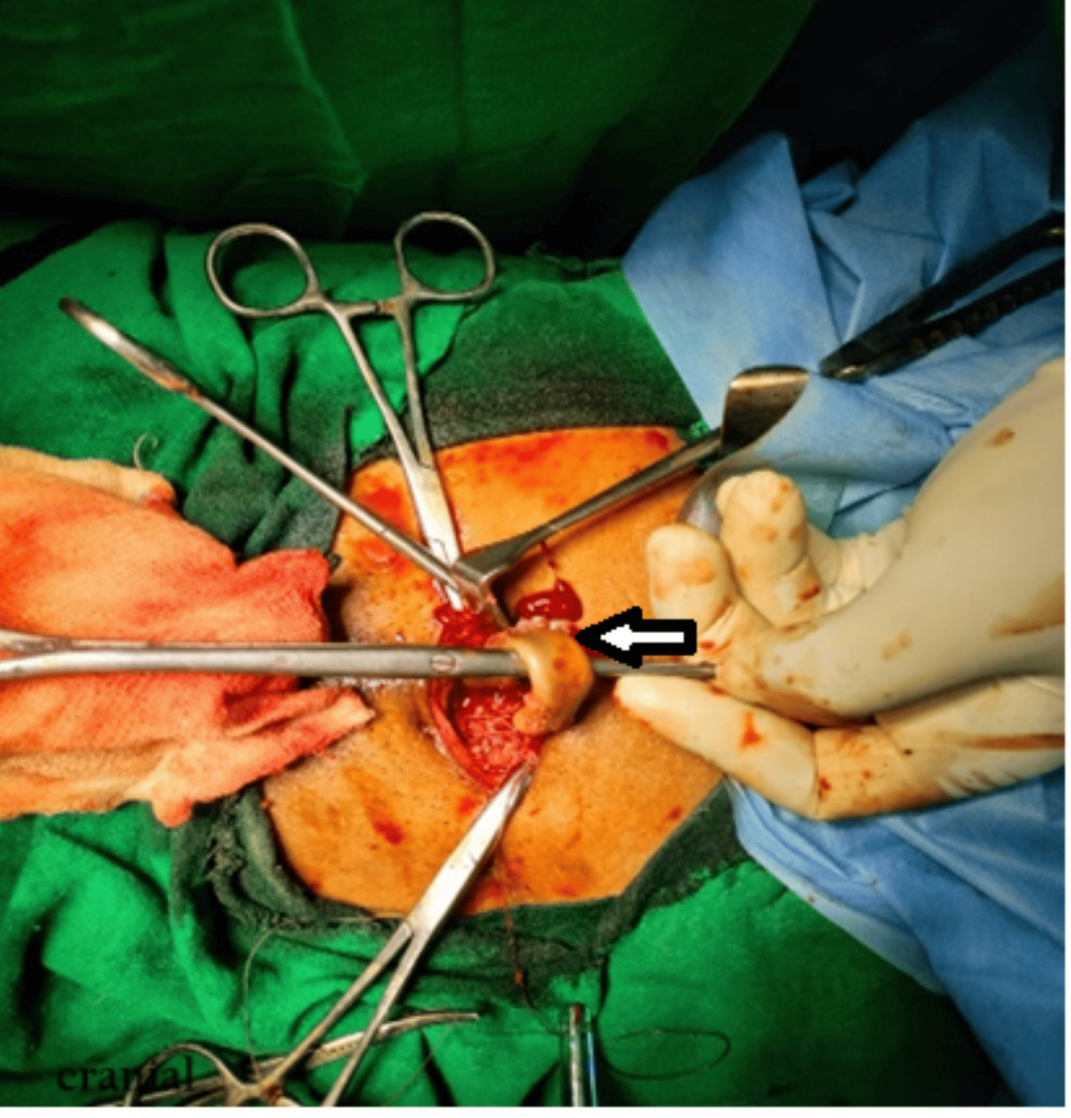
Intraoperative image showing the drain within the bladder (arrow)

**Figure 5:**
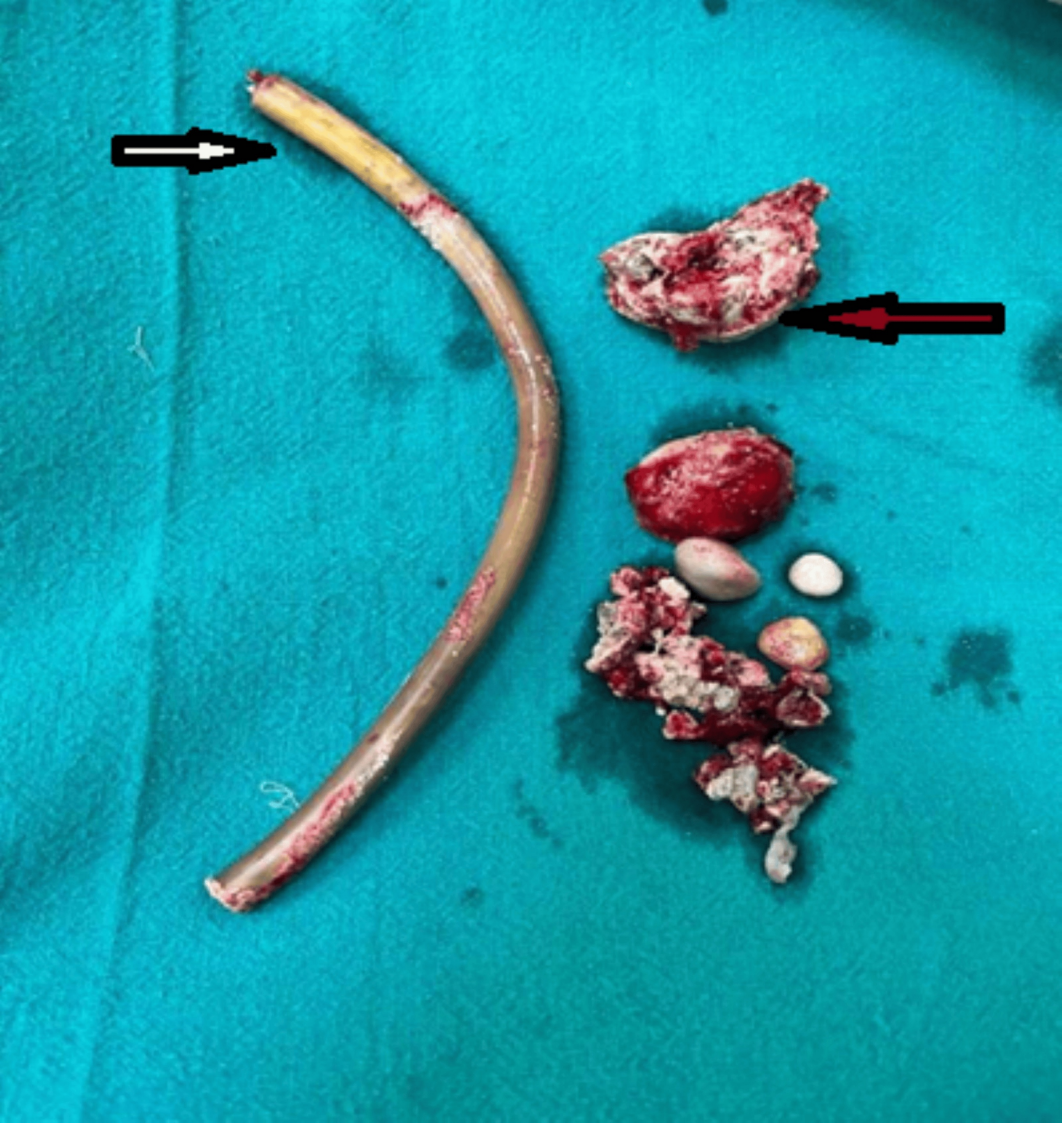
Postcystolithotomy specimen showing multiple stones (red arrow) and retained drain with crusting (white arrow)

The patient planned for exploration with Pfannenstiel incision and cystolithotomy. The patient had intraoperative findings of multiple vesical calculi with the largest measuring 4x3 cm, along with a retained drain of length 10 cm, which was removed from the bladder. Additionally, two stones, each measuring 2x1.5 cm, were removed from the penile urethra (Figures 4, 5).

Suprapubic cystostomy was done with a 16 F Foley catheter and the bladder was closed in two layers with Vicryl 2-0 simple continuous sutures. Per urethral Foley catheter (16 F) was inserted under direct vision.

The patient was discharged on postoperative day 4 with a suprapubic catheter (SPC) and per urethral foleys in situ. The urethral catheter was removed on postoperative day 14. The patient was doing well postoperatively and the kidney function test improved (blood urea 67mg/dl, creatinine 1.01 mg/dl). Voiding cystourethrogram was done via suprapubic cystostomy and a partial stricture at the level of the bulbo-membranous junction was identified; the patient planned for cystoscopy. The scope was negotiable beyond stricture (passable stricture), and urethral dilatation was done. The SPC was removed after one month of surgery.

## Discussion

Bladder stones comprise approximately 5% of all cases of urolithiasis, indicating a relatively small, yet significant, subset of this broader condition [3]. Secondary bladder stones form because of some underlying urinary tract pathology like bladder outlet obstruction (BOO), neurogenic bladder dysfunction, foreign bodies, bladder diverticula, and bladder augmentation or urinary diversion. BOO is currently the most common predisposing factor for bladder stone formation in the adult population and accounts for 45-79% of vesical calculi [4,5]. The incidence of bladder stone formation in neurogenic bladder due to spinal cord injury patients is 3.3%, with bladder augmentation it is 2-44% in adults, and in urinary diversion cases, the range is 0-3% [6-8]. Foreign bodies like misplaced double j stents, broken pieces of a drain, or sutures that accidentally pass through the bladder become encrusted and form nidus for stones causing secondary bladder stones. The patients usually present with features of atypical urinary tract symptoms like repeated urinary tract infection, painful voiding, hematuria, frequency, and voiding difficulty. Primary bladder stones usually do not cause obstructive renal injury until they become larger in size. Larger bladder stones can cause BOO leading to hydronephrosis. Foreign bodies in the bladder can cause unilateral hydronephrosis by blocking the ureterovesical junction [9]. The clinical presentation of periurethral abscess with hydronephrosis with azotemia with features suggestive of obstructive uropathy is very rare in cases of bladder stones.

After seeing the intraoperative findings, clinical history was again confirmed with the patient retrospectively and the history of suprapubic catheterization with retropubic drain placement was done for the patient during previous cystolithotomy surgery. The patient gave a history of removal of the SPC and the drain broke, whilst its removal was missed by the treating physician, the patient also failed to notice the same and it was left in situ. The broken piece of drain migrated to the bladder, traversed through the peri vesical region, and eroded the urethra forming a nidus for infection and stone formation. Following this, the patient started having difficulty in voiding with increased frequency and LUTS for which the patient did not seek consultation, which further led to the development of obstructive uropathy with renal injury and periurethral abscess formation.

## Conclusions

This case underscores the critical importance of meticulous follow-up and management in patients with urinary tract procedures. The rare occurrence of vesical stones secondary to a retained intravesical drain highlights a potential pitfall in surgical practice and emphasizes the need for thorough postoperative monitoring. The patient’s presentation of penile swelling, LUTS, and obstructive uropathy ultimately led to the discovery of multiple bladder stones and a retained drain, which were not initially recognized. Regular follow-up and patient education on potential symptoms of complications can further mitigate the risk of such rare but significant issues.
